# Evaluation of antibiotic susceptibility patterns of pathogens isolated from routine laboratory specimens at Ndola Teaching Hospital: A retrospective study

**DOI:** 10.1371/journal.pone.0226676

**Published:** 2019-12-23

**Authors:** Warren Chanda, Mespa Manyepa, Ephraim Chikwanda, Victor Daka, Justin Chileshe, Mathias Tembo, Joseph Kasongo, Allen Chipipa, Ray Handema, John A. Mulemena

**Affiliations:** 1 Mulungushi University, School of Medicine and Health Sciences, Livingstone, Zambia; 2 Tropical Diseases Research Centre, Ndola, Zambia; 3 Copperbelt University, School of Medicine, Ndola, Zambia; 4 Department Pathology, Ndola Teaching Hospital, Ndola, Zambia; Michigan State University, UNITED STATES

## Abstract

Periodic monitoring of antibiotic susceptibility patterns in clinical settings is vital to ascertain the potency as well as re-establishing empirical therapy. This retrospective study aimed to evaluate the antibiotic susceptibility patterns of pathogens isolated from routine laboratory specimens at Ndola Teaching Hospital. A retrospective study was conducted on routine specimens received between May 2016 and July 2018. Specimens were cultured on standard media and Kirby-Bauer disc diffusion method was used for susceptibility testing in accordance with the Clinical and Laboratory Standard Institute’s recommendations. A total of 693 specimens were analyzed, of which 65.9% (457) specimens came from inpatient departments and 49.1% (340) came from female patients. The commonest specimens were urine (58.6%), blood (12.7%) and wound swabs (8.5%), and the most common microorganisms were coliform (29.3%), *Staphylococcus aureus* (15.4%), coagulase negative *Staphylococci* (CoNS, 13.4%), and *Escherichia coli* (13%). The highest percentage of resistance to any particular antibiotic was co-trimoxazole (91.7%, 33) followed by nalidixic acid (75.2%, 279), norfloxacin (69.0%, 100), ceftazidime (55.7%, 185), nitrofurantoin (46.6%, 191), chloramphenicol (43%, 111) and ciprofloxacin (8.6%, 271). Furthermore, patient location had resistance effect on coliform (p = 0.014), CoNS (p = 0.031), *Streptococcus* species (p = 0.024) and *Klebsiella* species (p = 0.004) to nitrofurantoin, ceftazidime, nitrofurantoin and chloramphenicol, respectively. Besides coliform, resistance of *Enterobacter* species to ceftazidime and *Proteus* species to nalidixic acid were more from female patients. Generally, the most effective antibiotics were chloramphenicol and nitrofurantoin with addition of ceftazidime on blood pathogens and ciprofloxacin on wound swab pathogens. The common isolates were coliform, *S*. *aureus*, coagulase negative *Staphylococci* and *Escherichia coli*. The resistance of most bacteria to ceftazidime and nitrofurantoin were influenced by both gender and location. Our study presents a broad overview of the resistance profiles of bacterial isolates. However, more nosocomial prevalence and antibiogram studies on individual routine specimens are required to provide a more detailed picture of resistance patterns.

## Introduction

Microbial infectious diseases have a devastating effect on the well-being of humans. Fortunately, antibiotic agents came to the aid of alleviating human bacterial infections ever since penicillin was discovered in 1928 by Alexander Fleming [1]. Its utilization in the 1940s marked the genesis and proliferation of conventional antibiotic agents in medicine [1]. These agents received preference over natural compounds in infectious diseases management because of their greater effectiveness and selectivity [2]. However, their widespread usage in preventing and treating human, animal and plant infections led to the emergence and spread of antibiotic resistance due to selective pressure on susceptible strains causing the survival of resistance strains [1, 3].

Antibiotic resistance (AR) can be due to natural, acquired or clinical resistance and is the ability of bacteria (in this case, pathogenic bacteria) to resist the effect of antibiotic agents [4]. Natural or intrinsic resistance is an inborn phenomenon for bacteria to resist antibiotics without prior antibiotic exposure and without horizontal gene transfer whereas acquired resistance is as a result of intrinsic gene mutation with prior exposure to certain mutagens, antibiotic or through horizontal genetic exchange [5]. For instance, intrinsic genes for beta lactamases and multidrug efflux pumps encoded on the chromosomes perform different protective functions for bacteria such as involvement in the biosynthesis of the cell wall, trafficking of signalling molecules or detoxification of metabolic intermediates among others [6]. Moreover, the presence of mobile elements with associated AR genes such as plasmids, transposons, and integrons encourage the dissemination of antibiotic determinants amongst different bacteria resulting into acquired resistance [7]. There are several other aspects that contribute to antibiotic resistance like incorrect diagnosis, irrational use of antibiotics, and irregular antibiotic consumption possibly due to an incorrect prescription or to poor compliance [8]. Therefore, improving on these aspects can prevent the spread of antibiotic resistance.

On the other hand, nosocomial infections which are hospital acquired infections that occur within 72 hours after patient admission, pose a great challenge to the welfare of patient management [9, 10]. This ultimately increases the length of stay for in-patients and impacts negatively on hospital costs. Immunocompromised patients such as the elderly and children, patients with underlying diseases, those undergoing medical or surgical treatments, and antibiotic use and long-term care in hospitals contribute to the rapid emergence of nosocomial pathogens [11]. There is up to 70% chance for patients admitted to a room previously occupied by a patient with *Clostridium difficile*, *Pseudomonas aeruginosa*, methicillin-resistant *Staphylococcus aureus* (MRSA), *Acinetobacter baumannii* or vancomycin-resistant *Enterococci* (VRE) to obtain these microbes during hospital stay [12–14]. Moreover, nosocomial pathogens are resistant to at least one of the commonly used antibiotics in clinic settings and continued exposure of these pathogens to antibiotics increases resistance [15, 16]. A study by Zhang *et al*. (2013)[17] revealed that the antibiotic route of antibiotic administration influences the level of AR in gut microbiota, while commensal bacteria facilitate the spread of antibiotic resistance [16, 17]. Therefore, analysing the efficacy of various antibiotics used in a hospital setup is important. This can be achieved through periodic infection control practices (like microbial hygiene assessments) that may provide insights on how clean a surface is by sampling various surfaces such as taps, sinks, toilets, beds, and floors for epidemiological investigations to assess the spread of nosocomial pathogens and their associated antibiotic susceptibility patterns [18].

The escalating levels of hospital and community-acquired infections caused by antibiotic resistant pathogens have reduced the choices of implementing an effective antibiotic therapy [19]. Additionally, as the number of resistant strains increase in clinical settings, broad spectrum antibiotics become the ultimate choice, but the manifestation of resistance to broad spectrum antibiotics in multidrug resistant strains reduces the chances of choosing an effective empirical therapy [20]. While the prevailing crisis of AR has been reported elsewhere, the resistance status towards commonly used antibiotics at Ndola Teaching Hospital (NTH) is not yet known. There is a paucity of AR information in Zambia on commonly used antibiotics in hospitals and no such study has been conducted at NTH. This retrospective study aimed at evaluating the antibiotic susceptibility profiles of organisms isolated from routine specimens sent for culture at Ndola Teaching Hospital microbiology laboratory from May 2016 to July 2018.

## Materials and methods

### Study site

Ndola Teaching Hospital is a provincial referral hospital for Copperbelt, Luapula and North-western provinces of Zambia. The NTH microbiology laboratory participates in a bacteriology External Quality Assessment (EQA) program with Oneworld Accuracy Support through the Ministry of Health-Zambia and is working towards obtaining accreditation by the Southern African Development Community Accreditation Service (SADCAS).

### Data collection

This retrospective study was based on the Disa*Lab system (http://www.disalab.com) generated report on all isolated organisms in the NTH microbiology laboratory for 2 years from May 2016 when the system was commissioned to July 2018. The microbiology section of the laboratory receives several kinds of specimens from inpatient and outpatient departments for bacteriological analysis. The bacteriological analysis involves culturing of specimen on appropriate culturing media following the national standard operating procedures and Clinical and Laboratory Standards Institutes (CLSI) guidelines [21]. The isolated organism is further exposed to different identification tests using in-house and/or commercially prepared biochemical media such as Sulphur Indole Motility (SIM) agar (Becton, Dickinson and company [BD], USA), Triple Sugar Iron (TSI) agar (BD, USA), Lysine Iron Agar (BD, USA), Citrate agar (Mast Group Ltd, UK), urea media (BD, USA), oxidase reagent (Himedia, India), hydrogen sulphide (VYKing Pharmaceuticals Ltd, Zambia) or Analytical profile index (API) 20 for Enterobacteriaecae (bioMerieux^®^ SA, France). Further, the antibiotic susceptibility testing (AST) is performed using a Kirby-Bauer disc diffusion method on the isolated/identified organism by preparing the bacterial suspension in comparison with 0.5MacFarland turbidity standard and inoculating on Mueller-Hinton agar (BD, USA) or Blood supplemented Mueller-Hinton agar [21]. Quality control is performed with various standard strains such as *Staphylococcus aureus* (ATCC 25923), *Escherichia coli* (ATCC 25922), *Pseudomonas aeruginosa* (ATCC 27853), *Proteus mirabilis* (ATCC 12453), *Haemophilus influenza* (ATCC 49766) and *Enterococcus faecalis* (ATCC 29212). A Disa*Lab system generated Microsoft Excel spreadsheet report for microbiology laboratory on the ASTs that had been performed on all isolated organisms from May 2016 to May 2018 was analysed.

### Inclusion and exclusion criteria

All specimen entries having information on the age of a patient, source of specimen, type of specimen, isolated organism and ASTs performed was included in this study. However, entries without any of the aforementioned information or specimens with unknown specimen type, unknown source of specimen and/or specimen without organism was excluded from this study. Also, identified organisms having less than three entries were excluded from the study. Moreover, all partially identified lactose fermenting Gram negative bacilli were included in the study as coliforms (except *Escherichia coli*).

### Data analysis

The AST results were analysed with Microsoft Excel 2010 and IBM SPSS Statistics version 20 software. The rates of susceptibility for individual antibiotics was calculated for every bacterial isolate by age and gender of patient, specimen source (location), year of sample processing and type of specimen. The mean percentages of the susceptibility of each isolate to all tested antibiotics was calculated as the number of resistant strains out of the total number of strains exposed to a particular antibiotic in a specimen. Age and gender of patient, specimen source and type of specimen comparisons were performed using the Pearson Chi-square test and Fisher’s exact test after checking the applicability conditions and a p-value of ≤ 0.05 was considered significant.

### Ethical consideration

The ethical clearance was obtained from Tropical Diseases Research Centre Ethics Review Committee (TDR/C4/03/2019) and National Health Research Authority while permission to use the Disa*Lab system generated data was obtained from Ndola Teaching Hospital laboratory management. No personal identifiers were included in the study.

## Results

### The overall characterization of patient specimens and bacterial isolates

**Fig 1:**
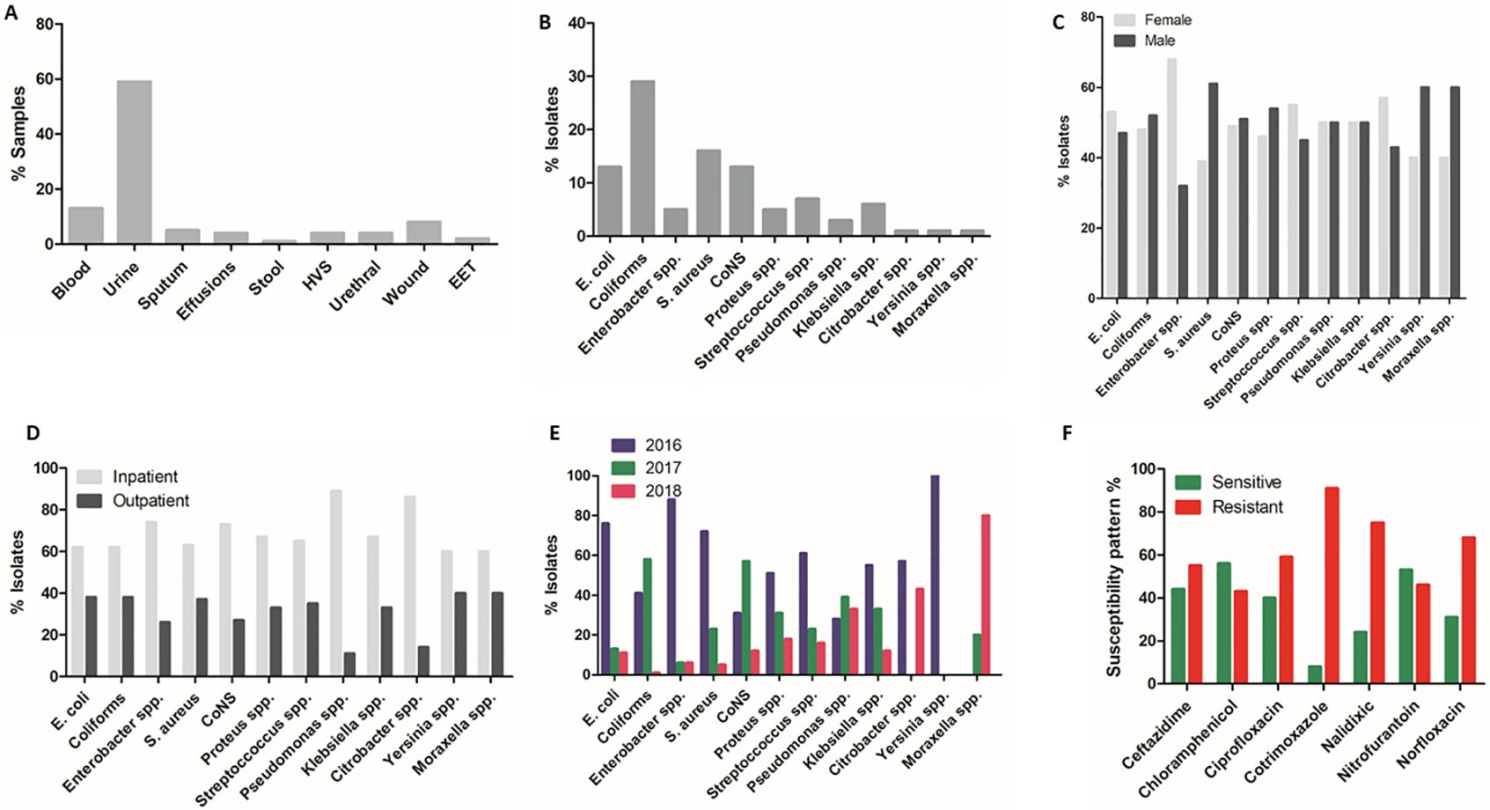
The overall percentage of bacterial isolates and their antibiotic susceptibility patterns. The overall percentage of specimens (A) and bacterial isolates (B), bacterial isolates between male and females (C), between inpatient and outpatients (D), the percentage of bacterial isolates from 2016 to July 2018 (E), and the overall potency of antibiotics on isolated bacteria (F). CoNS: coagulase negative Staphylococci, spp: species, Nalidixic: nalidixic acid, HVS: high vaginal swab, and EET: eye, ear and throat swabs.

A total of 2659 specimens were analyzed and only 693 (26%) met the inclusion criteria for this study. Of the 693, 340 (49.1%) were from female patients and 353 (50.9%) from male patients, ranging in age between 15 and 97 years with a mean average of 40.4 ± 17 years. 457 (65.9%) specimens were from inpatient department while 236 (34.1%) were from outpatient department, and the highest number of specimen type was urine (406/693) followed by 88/693 of blood (S1 Table; Fig 1A). Furthermore, 64 (9.2%) specimens were the lowest number received between January and July of 2018 while 2016 and 2017 received 375 (54.1%) and 254 (36.7%), respectively. Out of the total specimens (693), 52 (7.5%) came from patients younger than 20 years, 172 (24.8%) from patients between 20 and 29 years, 176 (25.4%) from patients between 30 and 39 years, while the high number of 293 (42.3%) was collected from patients of age 40 and above.

The commonest bacteria isolated from these specimens were coliform, *Escherichia coli*, *Staphylococcus aureus* and coagulase negative *Staphylococci* (Fig 1B). However, the picture of isolates between male and female patients were almost equal (Fig 1C) but more isolates from inpatients than outpatients (Fig 1D). The highest numbers of specimens for 2016 (375/693) and 2017 (254/693) correlated with the numbers of isolates identified in these year periods (Fig 1E) but the less numbers for 2018 were attributed to the lower number of specimens analyzed. Generally, isolates were resistant to cotrimoxazole (91%), nalidixic acid (75%), norfloxacin (68%) and ciprofloxacin (59%) although chloramphenicol (56%) and nitrofurantoin (53%) retained their effectiveness (Fig 1F); and this could be attributed to the frequency of specimen reception, bacterial isolate and the panel of antibiotics used (S1 Table, Fig 1A & 1F).

### The characterization of bacterial isolates from blood, urine and wound swab specimens

**Fig 2:**
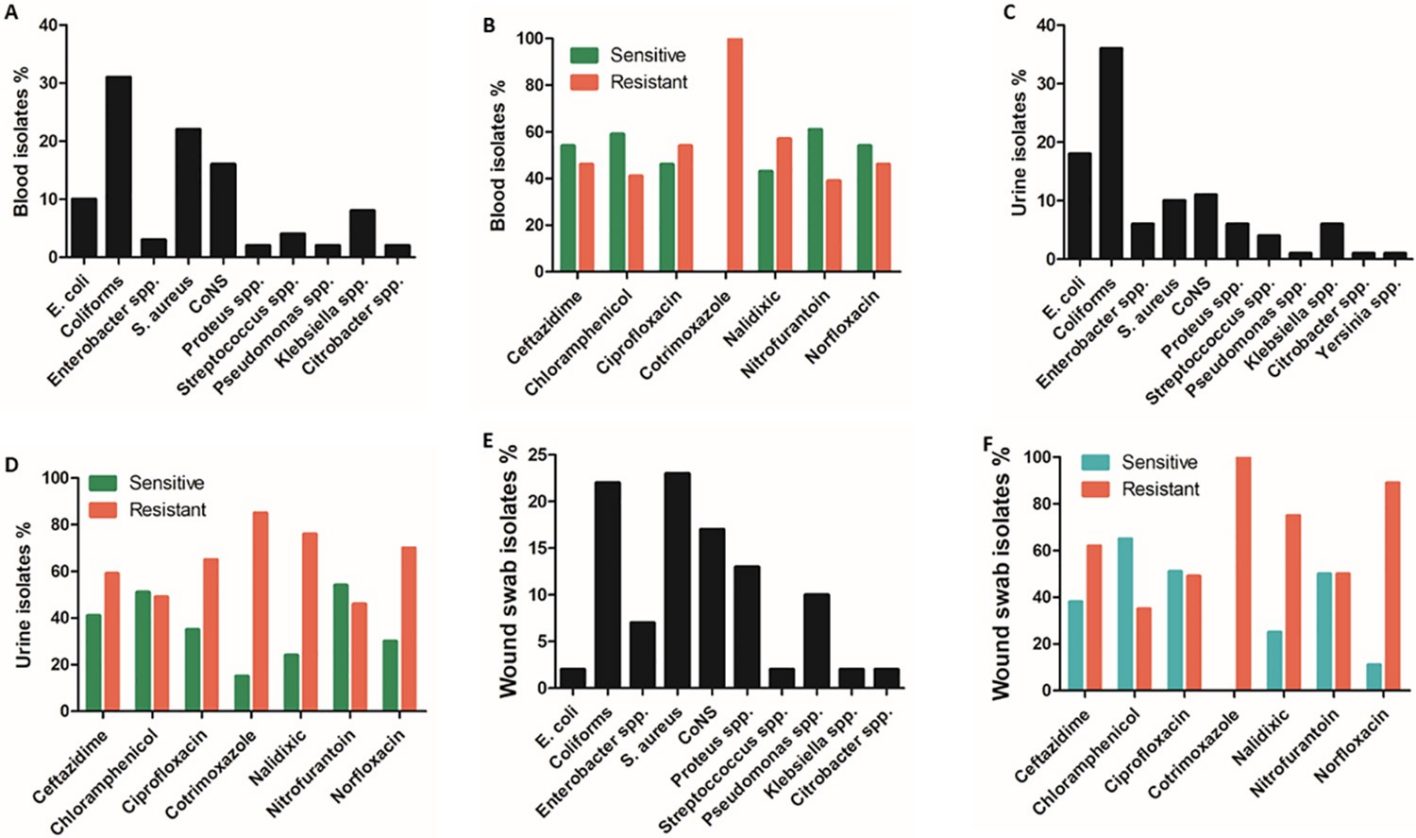
Characterization of bacterial isolates from blood, urine and wound swab specimens. The percentages of bacterial isolates from blood (A), urine (C) and wound swab (E) specimens; and the percentages of antibiotic susceptibility patterns of isolates from blood (B), urine (D) and wound swab (F) specimens. CoNS: coagulase negative *Staphylococci*.

Since the highest frequency of specimens received at NTH microbiology laboratory were blood, urine and wound swab specimens (Fig 1A); we thought to identify the common isolates from these specimens and their antibiotic susceptibility patterns. From blood specimens; coliform, *Staphylococcus aureus* and coagulase negative *Staphylococci* (CoNS) were the commonest isolates (Fig 2A) and overall, ceftazidime, chloramphenicol, nitrofurantoin and norfloxacin were 54%, 59%, 61% and 54% effective against blood pathogens, respectively (Fig 2B). The common uropathogens were coliform and *E*. *coli*, and the general picture revealed gross resistance to utilized antibiotics except for chloramphenicol and nitrofurantoin that retained potency by 51% and 54%, respectively (Fig 2C & 2D). Moreover, a wide range of pathogens were commonly isolated from wound swab specimens such as *S*. *aureus*, coliform, CoNS, *Proteus* species and *Pseudomonas* species (Fig 2E). From the list of antibiotics, wound swab isolates were resistant to all except chloramphenicol, ciprofloxacin and nitrofurantoin that were 65%, 51% and 50% effective, respectively (Fig 2F).

### The effect of age, gender and patient location on antibiotic susceptibility pattern

The prevalence of bacterial pathogens has an association with age and gender of patients (Magliano, 2012). Also, the location of the patient in the hospital may contribute to bacterial resistance levels. For example, about 70% patients admitted to a room previously occupied by a patient with *Clostridium difficile*, *Pseudomonas aeruginosa*, methicillin-resistant *Staphylococcus aureus* (MRSA), *Acinetobacter baumannii* or vancomycin-resistant *Enterococci* (VRE) are likely to obtain these microbes during hospital stay (Galvan, 2012; Huang, 2006; Nseir, 2011). Therefore, we wanted to understand the impact of age, gender and location of patient on antibiotic susceptibility patterns. In this study, the number of bacteria exposed to antibiotics was not homogenous. For instance, out of 693 bacterial isolates from various specimens included in this study, 332(48%) were exposed to ceftazidime, 457(66%) to chloramphenicol, 36(5%) to cotrimoxazole, 371(54%) to nalidixic acid, 410(59%) to nitrofurantoin, and 145(21%) to norfloxacin. We observed that coliform isolates from male patients were 43.2% resistant to nalidixic acid (p = 0.004), while *Enterobacter* species and *Proteus* species from female patients were more resistant to ceftazidime (42.9%, p = 0.017) and nalidixic acid (54.2%, p = 0.028), respectively (Table 1). Also, as presented in Table 1, most coliform, CoNS, *Streptococci* and *Klebsiella* species isolated from inpatient departments were resistant to nitrofurantoin, ceftazidime and chloramphenicol.

**Table 1:** The resistance patterns of some bacteria with respect to patient location, gender and age group.

**Gender**				
**Microorganism**	**Female %(n)**	**Male %(n)**	**p value**	**Drug**
**Coliform**	26.8 (40/149)	43.2 (65/149)	0.004	Nalidixic acid
***Enterobacter* species**	42.9 (9/21)	0 (0/21)	0.017	Ceftazidime
***Proteus* species**	54.2 (13/24)	20.8 (5/24)	0.028	Nalidixic acid
**Location**				
**Microorganism**	**Inpatient %(n)**	**Outpatient %(n)**	**p value**	**Drug**
**Coliform**	28.1 (39/139)	12.2 (17/139)	0.014	Nitrofurantoin
**CoNS**	40.7 (11/27)	7.5 (2/27)	0.031	Ceftazidime
***Streptococcus* species**	30 (6/20)	0 (0/20)	0.024	Nitrofurantoin
***Klebsiella* species**	45 (9/20)	0 (0/20)	0.004	Chloramphenicol

CoNS: coagulase negative *Staphylococci*, n: number, %: percent.

Furthermore, we thought to understand the effectiveness of antibiotics on isolates from blood, urine and wound swab specimens with regards to age, gender and location of patients. According to the univariate tests analysis, age (p>0.05) and gender (p>0.05) had no effect on the effectiveness of used antibiotics on all isolates. However, patient location (i.e. inpatient or outpatient) contributed to the susceptibility pattern of ceftazidime (Fig 1F; Table 2) and nitrofurantoin (Fig 1F; Table 2) on all isolates. Considering isolates from blood, urine and wound swab specimens as the frequently isolated bacteria (Fig 1A), age showed no effect on the susceptibility patterns of used antibiotics (p>0.05). But, location of a patient had an effect on the potency of ceftazidime on bacteria from blood, urine and wound swab specimens; and on the potency of nitrofurantoin and ciprofloxacin on bacteria from urine and wound swab specimens respectively (Table 2). The differences on the potency of ceftazidime and ciprofloxacin on wound swab specimen isolates were 21.3% (Eta^2^ = 0.213) and 17.2% (Eta^2^ = 0.172) respectively between inpatients and outpatients (Table 2). We further noticed that effectiveness of ciprofloxacin on uropathogens was hampered by gender (p = 0.033) with a minute difference of 1.8% (Eta^2^ = 0.018) between male and female patients as presented in Table 2.

**Table 2:** The effects of gender and location of patient on the efficacy of antibiotics.

Type of Isolates	Dependent factor	Independent factor	P value	Partial Eta Squared
Overall	Ceftazidime	Location	0.001	0.032
	Nitrofurantoin	Location	0.025	0.013
Blood	Ceftazidime	Location	0.049	0.198
Urine	Ceftazidime	Location	0.015	0.029
	Ciprofloxacin	Gender	0.033	0.018
	Nitrofurantoin	Location	0.051	0.013
Wound	Ceftazidime	Location	0.047	0.213
	Ciprofloxacin	Location	0.028	0.172

### The antibiotic resistance of bacterial isolates from blood, urine and wound specimens

As Table 2 highlights the total effects of location and gender of patient on various antibiotics, we wanted to identify the isolate that was mostly resistant. Amongst bacteremia causative agents, coliform (27), *S*. *aureus* (19), CoNS (14) and *E*. *coli* (9) were frequently isolated (Table 3A). The lower number of individual isolates being exposed to particular antibiotic hampered proper identification of the most resistant bacterium. However, among the Gram negative bacteria, coliform were susceptible to all except co-trimoxazole, and *E*. *coli* was susceptible to ceftazidime whereas the Gram positive bacteria (*S aureus* and CoNS) were susceptible to chloramphenicol (Table 3A). The potency of ceftazidime was high on blood specimen isolates with a 19.8% (Eta^2^ = 0.198, Table 2) difference between inpatients and outpatients because the drug was highly tested (541%, Table 3A) on isolates from blood specimens.

**Table 3:** (A) Antibiotic resistance profiles of bacteria from blood specimens. (B) Antibiotic resistance profiles of bacteria from urine specimens. (C) Antibiotic resistance profiles of bacteria from wound swab specimens.

Microorganisms		Antibiotic agents						
	n	CAZ %(n)	C %(n)	CIP %(n)	SXT %(n)	NA %(n)	NIT %(n)	NOR %(n)
(A)								
*E*. *coli*	9	40 (2)	66.7(4)	57.1(4)	100(1)	66.7(4)	42.9(3)	33.3(1)
Coliform	27	30(3)	46.2(6)	43.8(7)	100(2)	44.4(4)	44.4(4)	0
*Enterobacter* species	3	0	100(1)	0	100(1)	0	0	ND
*S*. *aureus*	19	75(3)	37.5(3)	63.6(7)	ND	100(1)	0	100(1)
CoNS	14	100(1)	25(3)	55.6(5)	100(1)	100(1)	33.3(1)	ND
*Proteus* species	2	0	0	100(1)	ND	100(2)	100(1)	100(1)
*Streptococcus* species	3	100(1)	100(1)	66.7(2)	ND	ND	100(1)	ND
*Pseudomonas* species	2	ND	0	100(1)	ND	ND	100(1)	ND
*Klebsiella* species	7	50(1)	0	33.3(2)	100(1)	33.3(1)	33.3(1)	33.3(1)
*Citrobacter* species	2	ND	100(2)	100(2)	100(1)	ND	ND	100(1)
Total	88	54.1(11)	20.5(18)	35.2(31)	8.0(7)	14.8(13)	13.6(12)	5.7(5)
(B)								
*E*. *coli*	73	61.4(27)	25.9(7)	62.5(35)	100(3)	75.9(41)	47.5(29)	61.5(8)
*Coliform*	147	50(39)	66.7(20)	60.8(59)	100(1)	71.2(89)	38.1(45)	71.7(43)
*Enterobacter* species	23	46.2(6)	87.5(7)	72.7(8)	50(1)	75(12)	66.7(12)	100(1)
*S*. *aureus*	40	92(23)	36.8(7)	64.7(22)	ND	92.3(24)	37.5(12)	75(3)
CoNS	44	40(6)	25(4)	70(14)	100(2)	87.5(21)	43.3(13)	83.3(5)
*Proteus* species	25	44.4(4)	83.3(5)	62.5(10)	100(3)	77.8(14)	66.7(14)	50(2)
*Streptococcus* species	16	62.5(5)	75(3)	66.7(6)	ND	100(9)	36.4(4)	71.4(4)
*Pseudomonas* species	4	33.3(1)	100(1)	50(1)	ND	100(2)	50(2)	66.7(2)
*Klebsiella* species	25	69.2(9)	44.4(4)	68.4(13)	0 (0)	56.2(9)	75(12)	40(2)
*Citrobacter* species	4	75(3)	50(1)	100(4)	100(1)	100(3)	66.7(2)	100(1)
*Yersinia* species	5	100(4)	66.7(2)	100(4)	ND	60(3)	25(1)	100(1)
Total	406	31.2(127)	15.0(61)	43.3(176)	2.7(11)	55.9(227)	36.0(146)	18.0(73)
(C)								
*E*. *coli*	1	50(4)	ND	100(1)	ND	ND	0	100(3)
Coliform	13	ND	33.3(1)	50(4)	ND	83.3(5)	75(3)	ND
*Enterobacter* species	4	100(3)	0	100(1)	ND	100(2)	100(3)	ND
*S*. *aureus*	14	57.1(4)	0	40(4)	100(2)	100(3)	100(4)	66.7(2)
CoNS	10	75(3)	0	66.7(4)	100(1)	100(1)	100(3)	100(1)
*Proteus* species	8	25(1)	50(1)	40(2)	ND	100(3)	100(1)	100(2)
*Streptococcus* species	1	100(1)	100(1)	100(1)	ND	ND	100(1)	ND
*Pseudomonas* species	6	100(2)	100(3)	0	100(1)	100(1)	100(1)	ND
*Klebsiella* species	1	ND	100(1)	100(1)	ND	ND	ND	ND
*Citrobacter* species	1	ND	0	0	ND	ND	ND	ND
Total	59	30.5(18)	11.9(7)	30.5(18)	6.8(4)	25.4(15)	27.1(16)	13.6(8)

ND: not done, CAZ: Ceftazidime, C: Chloramphenicol, CIP: Ciprofloxacin, SXT: Co-trimoxazole, NA: Nalidixic acid, NIT: Nitrofurantoin, NOR: Norfloxacin, n: number, %: percent

Globally, urinary tract infections are among the most common community-acquired infections and *Escherichia coli* is the most common pathogen [22]. During the period under review, there were more urine specimens received at NTH laboratory with coliform isolates. Due to the incomplete identification of lactose fermenting Gram negative bacilli (coliform), we decided to assess the resistance patterns of all isolates from urine specimens to ascertain the resistance patterns of uropathogens. As presented in Table 3B, the most common pathogens were coliform (147), *E*. *coli* (73), *S*. *aureus* (40), *Klebsiella* (25), *Proteus* (25) and *Enterobacter* species (23), and almost all isolated organisms showed resistance to the used panel of antibiotics. The drugs with less potency as shown in Fig 2D were co-trimoxazole (84.6%, 11/13), nalidixic acid (76.2%, 227/298), norfloxacin (69.5%, 73/105), ciprofloxacin (64.7%, 176/272), ceftazidime (58.8%, 127/216), chloramphenicol (48.8%, 61/125) and nitrofurantoin (45.9%, 146/318). Among the AR-tested bacterial isolates, ciprofloxacin, co-trimoxazole, nalidixic acid and norfloxacin were less effective while on average, chloramphenicol, nitrofurantoin and ceftazidime were more effective against most uropathogens (Table 3B, Fig 2D).

Wound infection poses a serious challenge in clinical practice and usually lead to sepsis, limb loss, long hospital stays, higher costs, and contribute to increasing levels of human mortality and morbidity across the globe [23, 24]. Therefore, identifying the common isolates and their susceptibility patterns may aid in proper management of wound infections. At NTH, a total of 59 bacterial isolates were identified and among these, *S*. *aureus* (14), coliform (13), CoNS (10), *Proteus* species (8), *Pseudomonas* species (6) and *Enterobacter* species (4) emerged as the most common isolates (Table 3C). All isolates showed resistance to almost all antibiotics used with the exception of ciprofloxacin and chloramphenicol (Table 3C, Fig 2F).

## Discussion

In the recent past, antibiotic resistance (AR) has attracted attention in clinical settings worldwide due to its effects on increasing health-care costs, morbidity and mortality of patients from infectious diseases. The effect is even worsened in developing countries as information pertaining to antibiotic susceptibility patterns of bacterial isolates are sporadic [25]. However, it is worth mentioning that some important factors that encourage the dissemination of antibiotic resistance include the overuse/misuse of antibiotics due to factors such as incorrect diagnosis and the irrational use of antibiotics accounts for almost half of all antibiotic prescriptions for patients [8, 25]. Evidence based practice is still in its infancy in developing countries. Antibiotics are prescribed without laboratory analyses such as identifying the etiologic agent, antibiotic susceptibility testing, and/or testing for the presence of particular resistance markers. Similarly, several antibiotics are easily accessible over the counter in several pharmaceutical stores—which further increases the risk of emerging antibiotic resistance being witnessed today. Therefore, there is urgent need, especially in developing countries like Zambia to fight the spread of antibiotic resistance by instituting evidence based antibiotic therapy through increased access to diagnostic laboratories, promoting rational use of antibiotics in hospitals, strengthening AR surveillance programs, and educating the public, clinicians, pharmacists and veterinarians on the proper use of antibiotic drugs [25].

In a quest to understand the trend of AR, the current retrospective study was conducted to evaluate the antibiotic susceptibility profiles of organisms isolated from routine specimens sent for bacteriological culture at Ndola Teaching Hospital microbiology laboratory from May 2016 to July 2018. Generally, during the period of study, it was observed that urine, blood and wound swabs specimens were the commonest routine samples analyzed at NTH, of which the majority came from inpatient departments and from patients aged ≥40years (Fig 1A & 1D). From these specimens, coliform, *E*. *coli*, *S*. *aureus* and CoNS were the commonest pathogens encountered (Fig 1B), and chloramphenicol and nitrofurantoin proved to be effective during the period under study (Fig 1F). However, the frequency distribution of bacterial isolates on individual specimens were quite low (S1 Table) and this could have contributed to the general resistance observed. Moreover, the number of antibiotics used in susceptibility testing was not consistent, making it difficult to ascertain the cause of antibiotic resistance. Thus, antibiogram studies should be encouraged to help in implementing a systematic antibiotic resistance monitoring method.

Urine specimens dominated over other routine specimens (Fig 1A), and coliform, *E*. *coli*, *Klebsiella* species, *Proteus* species and *Enterobacter* species were the most isolated bacteria (Table 3B), and these findings were in agreement with other respective studies conducted elsewhere [26–30]. Urinary tract infections (UTIs) are the commonest reported bacterial infections in long term care facilities and this leads to increased use of antibiotics of which 50% of cases are believed to signify asymptomatic bacteriuria [31]. The trend of routine screening of asymptomatic bacteriuria is injurious to the patient as it increases the rates of recurrent infections with drug resistant bacteria due to increased selective pressure [32, 33]. In the current study, the increased number of urine specimens from inpatients (Fig 1D) may be the result of routine screening of asymptomatic bacteriuria perhaps due to the increased number of student interns working in these inpatient departments at NTH but the trend could reduce if or when UTI routine screening targets symptomatic patients. With the quest to understand the resistance patterns amongst uropathogens. We observed that the potency of ciprofloxacin, co-trimoxazole, nalidixic acid and norfloxacin had reduced amongst uropathogen isolates (Table 3B, Fig 2D) with an average resistance ranging from 58.8%– 84.6%. It was further observed that on average, the isolated uropathogens were susceptible to chloramphenicol, nitrofurantoin and norfloxacin, with nitrofurantoin having a low resistance rate (45.9%). In Nigeria, the uropathogenic *E*. *coli* was reported with resistance rates ranging from 51.1%–94.3% to most antibiotics with the exception of nitrofurantoin [30]. In other studies, Beyene *et al* (2011) found ciprofloxacin as the most effective drug against uropathogens and chloramphenicol as less sensitive while Shill *et al* (2010) revealed nitrofurantoin and cephalosporins as sensitive but not ciprofloxacin and amoxicillin on uropathogens isolated from diabetic patients [26, 28]. These studies and our current study suggest that nitrofuran drugs are still effective against uropathogens but calls for close monitoring since patient location (inpatient and outpatients) may have an effect on the sensitivity of ceftazidime (p = 0.029) and nitrofurantoin (p = 0.051) on uropathogens, and gender on ciprofloxacin (p = 0.033; Table 2) as observed in this study.

Septicemia is an important complication in health settings in developing countries and contributes to the rising number of morbidity and mortality. The current study showed that *S*. *aureus*, CoNS and coliform were the most isolated bacteria in blood specimens (Table 3A) and this was consistent with Ghadiri *et al*. (2012) study where CoNS and *E*.*coli* were the most cause of nosocomial bloodstream infections and UTIs, respectively [34]. Bloodstream pathogens were susceptible to ceftazidime and chloramphenicol. The effectiveness of ceftazidime was affected by patient location (p = 0.049) with 19.8% (Eta^2^ = 0.198) difference between inpatient and outpatient departments (Table 2). On the other hand, wound infection is another type of infection that poses a serious challenge in clinical practice and usually lead to sepsis, limb loss, long hospital stays, higher costs, and contribute to increasing levels of human mortality and morbidity across the globe [23, 24]. Among wound swabs pathogens, *S*. *aureus*, coliform, CoNS, *Proteus* species, *Pseudomonas* species and *Enterobacter* species were the commonest isolates, and were mostly sensitive to ciprofloxacin and chloramphenicol (Table 3C, Fig 2F). Moreover, the effectiveness of ciprofloxacin (p = 0.028) and ceftazidime (p = 0.047) on wound pathogens were affected by patient location, and the difference between inpatients and outpatient departments on the effectiveness of ciprofloxacin and ceftazidime was 17.2% (Eta2 = 0.172) and 21.3% (Eta2 = 0.213), respectively (Table 3). The prevalence of wound swab pathogens and the antibiotic susceptibility pattern in this study is in agreement with other studies conducted elsewhere [23, 24].

Although there were minor differences on the prevalence of bacterial species from male and female patients (Fig 1C), inpatient department recorded the highest number of isolates than outpatient departments (Fig 1D). This indicated the need to perform surveillance studies on nosocomial infections and establish the source of infection as this could not be established in the current study. Literature reveals that pathogens such as *Enterococcus faecium*, *Staphylococcus aureus*, *Klebsiella pneumoniae*, *Acinetobacter baumannii*, *Pseudomonas aeruginosa*, *and Enterobacter* species are commonly associated with multidrug resistance in nosocomial infections [35] and were the commonly isolated bacteria (except *A*. *baumanii*) in our study.

In addition, as presented in Table 1, most CoNS (40.7%), *Streptococci* (30%) and *Klebsiella* species (45%) isolates from inpatient department were resistant to ceftazidime (p = 0.031), nitrofurantoin (p = 0.024) and chloramphenicol (p = 0.004), respectively. Except for coliform isolates, *Enterobacter* species and *Proteus* species from female patients were more resistant to ceftazidime (p = 0.017) and nalidixic acid (p = 0.028). These findings were contrary to a 5 year study of McGregor *et al* (2013) which revealed minor differences of used drug susceptibility between male and female patients [36]. The study focused on the antibiotic resistance patterns of *E*. *coli* urinary isolates from outpatients while our current study focused on all routine specimen isolates from both inpatient and outpatient data. However, it is imperative to note that information from both male and female should be considered when designing empiric antibiotic therapy. Additionally, antibiogram studies are required on all inpatient department isolates as it will assist in instituting proper infection control measures to curb the scourge of antibiotic resistance.

Furthermore, other specimens such as sputum recorded *Streptococcus* species as the most common isolates and *Streptococcus pyogenes* was the most frequently isolated species (S1 Table). The commonest isolates in stool specimens were *S*. *aureus* and coliform; high vaginal swab (HVS) specimens recorded *S*. *aureus*, *Streptococcus* species, *E*. *coli* and coliform; body effusion specimens that included synovial, peritoneal, pleural, pericardial and cerebral spinal fluid had CoNS, *S*. *aureus* and *Streptococcus* species as the commonest isolates; urethral swabs recorded *S*. *aureus* and CoNS while swab specimens from the ears, eyes and throat were mostly affected by *S*. *aureus*, CoNS, *Klebsiella* species and coliform (S1 Table). These findings were in agreement with several other studies performed elsewhere [37–43] on similar type of specimens but Gram positive species such as coagulase negative *Staphylococci* from urethral swab specimens as observed in the current study may be as a result of skin contaminants, hence caution must be observed to avoid over diagnosis. As this may lead to increased resistance rates among bacterial isolates in clinical settings [8, 25].

However, we observed that most *Klebsiella* species, *Streptococcus* species and CoNS from inpatient departments were more resistant to chloramphenicol, nitrofurantoin and ceftazidime respectively than outpatient department isolates. Similarly, *Enterobacter* species and *Proteus* species from female patients were more resistant to ceftazidime and nalidixic acid, respectively than those from male patients but only coliform from males were more resistant to nalidixic acid.

## Conclusion

Generally, the most effective antibiotics were chloramphenicol and nitrofurantoin with addition of ceftazidime on blood pathogens and ciprofloxacin on wound swab pathogens. The common isolates were coliform, *S*. *aureus*, coagulase negative *Staphylococci* and *Escherichia coli*, but *Proteus* and *Pseudomonas* species were among the common isolates on wound swab specimens. The resistance of most bacteria to ceftazidime and nitrofurantoin were influenced by both gender (more for female patients) and location (more for inpatient departments). However, *E*. *coli* was the common uropathogen and was sensitive to nitrofurantoin. Since antibiotic resistance is the global issue and more problematic in developing countries where data is scarce, instituting surveillance programs to determine the prevalence of several resistant pathogens will help in managing patient care in clinical settings.

The data presented here are valuable and useful in a setting where information on antibiotic resistance is scarce. However, there were some limitations that needed to be addressed. First, the number of isolates that were exposed to some drugs were as little as one and the use of antibiotics was not consistent across all isolates as this could overstress the resistance picture. Second, there was no systematic way of collecting clinical specimens for microbiological cultures as this depended largely on the clinician’s judgement which could have introduced sampling bias. Third, the assumption that some samples were collected after starting an antibiotic therapy cannot be ignored, which may lead to over-representation of resistant isolates, and the relationship between in vitro potency of antibiotic agents and clinical potency in patients’ diseases could not be assessed because of the retrospective pattern of the study. Fourth, the susceptibility patterns were performed with the disc diffusion method which was not confirmed by a more accurate method like E-test or microbroth dilution method. Fifth, some antibiotic agents such as ampicillin, cefotaxime, cefuroxime, imipenem, meropenem, erythromycin, gentamycin, kanamycin, oxacillin, cefuroxime, penicillin G, Streptomycin, and vancomycin which are commonly available in Zambia were not tested during the study period, thus their susceptibility patterns at NTH remains unknown. Lastly, presumptive information on the prevalence of vancomycin resistant *Enterococci* (VRE), methicillin resistant *Staphylococcus aureus* (MRSA), carbapenemase and extended spectrum beta lactamase (ESBL) producing bacteria could not be established.

## Supporting information

S1 Table The frequency of bacterial isolates from various specimens. (DOCX)
