# Supplementary material for: Evaluation of antibiotic susceptibility patterns of pathogens isolated from routine laboratory specimens at Ndola Teaching Hospital: A retrospective study
Source: PLoS One. 2019 Dec 23;14(12):e0226676. doi: 10.1371/journal.pone.0226676 (PMC6927611; doi:10.1371/journal.pone.0226676)
Supplement: S1 Table — (DOCX) [file pone.0226676.s001.docx]

**Table S1:** The frequency of bacterial isolates from various specimens

| **Organism** | **Specimen** | | | | | | | | | Total  %(n) |
| --- | --- | --- | --- | --- | --- | --- | --- | --- | --- | --- |
|  | Blood  %(n) | Urine  %(n) | Sputum  %(n) | Effusions  %(n) | Stool  %(n) | HVS  %(n) | Urethral swabs  %(n) | Wound swabs  %(n) | EET swabs  %(n) |  |
| *E. coli* | 10.0(9) | 81.1(73) | 1.1(1) | 2.2(2) | 0(0) | 4.4(4) | 0(0) | 1.1(1) | 0(0) | 100.0(90) |
| Coliform | 13.3(27) | 72.4(147) | 2.5(5) | 0.5(1) | 1.0(2) | 2.0(4) | 1.0(2) | 6.4(13) | 1.0(2) | 100.0(203) |
| *Enterobacter* species. | 8.8(3) | 67.6(23) | 0(0) | 5.9(2) | 0(0) | 2.9(1) | 2.9(1) | 11.8(4) | 0(0) | 100.0(34) |
| *S. aureus* | 17.6(19) | 37.0(40) | 1.9(2) | 6.5(7) | 1.9(2) | 5.6(6) | 13.0(14) | 13.0(14) | 3.7(4) | 100.0(108) |
| CoNS | 15.1(14) | 47.3(44) | 4.3(4) | 9.7(9) | 0(0) | 3.2(3) | 5.4(5) | 10.8(10) | 4.3(4) | 100.0(93) |
| *Proteus* species*.* | 5.1(2) | 64.1(25) | 0(0) | 0(0) | 2.6(1) | 5.1(2) | 2.6(1) | 20.5(8) | 0(0) | 100.0(39) |
| *Streptococcus* species. | 6.1(3) | 32.7(16) | 34.7(17) | 8.2(4) | 2.0(1) | 8.2(4) | 4.1(2) | 2.0(1) | 2.0(1) | 100.0(49) |
| *Pseudomonas* species. | 11.1(2) | 22.2(4) | 11.1(2) | 11.1(2) | 0(0) | 0(0) | 5.6(1) | 33.3(6) | 5.6(1) | 100.0(18) |
| *Klebsiella* species*.* | 16.7(7) | 59.5(25) | 4.8(2) | 4.8(2) | 0(0) | 7.1(3) | 0(0) | 2.4(1) | 4.8(2) | 100.0(42) |
| *Citrobacter* species. | 28.6(2) | 57.1(4) | 0(0) | 0(0) | 0(0) | 0(0) | 0(0) | 14.3(1) | 0(0) | 100.0(7) |
| *Yersinia* species. | 0(0) | 100.0(5) | 0(0) | 0(0) | 0(0) | 0(0) | 0(0) | 0(0) | 0(0) | 100.0(5) |
| *Moraxella* species. | 0(0) | 0(0) | 80.0(4) | 0(0) | 0(0) | 0(0) | 0(0) | 0(0) | 20.0(1) | 100.0(5) |
| **Total** | **12.7(88)** | **58.6(40)** | **5.3(37)** | **4.2(29)** | **0.9(6)** | **3.9(27)** | **3.8(26)** | **8.5(59)** | **2.2(15)** | **100.0(693)** |
